# Relevance of Routine Postoperative Biochemical Tests in Primary Hip and Knee Arthroplasty Patients: Assessing Necessity and Clinical Outcomes

**DOI:** 10.7759/cureus.94782

**Published:** 2025-10-17

**Authors:** Raja Muhammad Mussab, Aiman Jawad, Rida Sohail, Mohammad Kayum, Mohamed El Taher, Shehanshah Muhammed Arqam

**Affiliations:** 1 Orthopaedics and Trauma, Jinnah Postgraduate Medical Centre, Karachi, PAK; 2 Orthopaedics and Trauma, Russell Hall Hospital, Dudley, GBR

**Keywords:** biochemical evaluation, blood test, eras protocols, hip and knee primary and revision arthroplasty, same day discharge

## Abstract

Introduction

Routine postoperative blood tests are common after primary hip and knee arthroplasty, but their value for every patient is uncertain in the era of enhanced recovery and routine transexamic acid. In the context of the United Kingdom (UK), this study sought to evaluate the clinical utility of next-day laboratory testing and to identify preoperative features that might permit selective testing.

Methods

A retrospective audit was performed in 99 consecutive primary total hip (n=51) and knee (n=48) arthroplasties undertaken at a tertiary centre. Demographic and laboratory data (pre- and post-operative haemoglobin, white cell count, platelets, electrolytes and creatinine/estimated glomerular filtration rate (eGFR)) were collected. Normality was assessed, and paired comparisons used a paired t-test or Wilcoxon signed-rank test as appropriate. Outcomes included blood transfusion, and other interventions prompted by test results, delays to discharge and readmissions. Statistical significance was set at p<0.05, and 95% confidence intervals were reported for mean differences.

Results

The cohort had a mean age of 69.5±10.1 years and comprised 50 men and 49 women. Mean haemoglobin fell from 136.33±12.83 g/L preoperatively to 115.81±13.03 g/L postoperatively. Mean haemoglobin change is 20.52 g/L (paired t=18.59; p<0.001), showing a statistically significant fall in haemoglobin after surgery. Mean white cell count rose and mean platelet count fell, consistent with an expected postoperative response. A total of 93 (93.9%) patients had normal renal indices, and 91 (91.9%) had normal electrolytes postoperatively. Moreover, nine patients (9.1%) were readmitted for clinical issues (wound problems, limb swelling or suspected venous thromboembolism (VTE) , infection or falls). No patient required blood transfusion. A total of 57 discharges (51.4%) were delayed while awaiting laboratory results.

Conclusion

Routine next-day blood tests after uncomplicated primary lower-limb arthroplasty produced predictable but infrequently actionable abnormalities and contributed to discharge delays. A selective, risk-based approach, directed at patients with low preoperative haemoglobin, impaired renal function, electrolyte derangement or other clinical concerns, appears safe and may reduce unnecessary testing, costs and length of stay.

## Introduction

Postoperative blood tests remain routine after major orthopaedic operations in many settings. These tests serve as a safeguard to identify complications early, especially in environments where the medico-legal climate encourages caution [1]. In response to enhanced recovery practices and agents like transexamic acid, the assumption that every patient requires routine postoperative tests is being questioned [2], even after elective total hip arthroplasty (THA) or total knee arthroplasty (TKA).

THA and TKA represent common, effective treatments for advanced osteoarthritis and inflammatory joint disease [3]. The standard practice after these uncomplicated primary procedures often includes a full blood count and urea and electrolytes for most patients, regardless of individual risk. This adds direct costs to services and potential delays in discharge while results are awaited [4].

In the United Kingdom (UK), hospital discharge after a total hip or knee arthroplasty has improved to same-day discharge (SDD) or a hospital stay of one to three days, with the help of enhanced recovery after surgery (ERAS) pathways [5]. Perioperative care has improved substantially over the last decade, as ERAS pathways support earlier mobilisation, shorter hospital stays and an ambition towards same-day arthroplasty where appropriate. Those changes create pressure to remove low-value processes that impede rapid discharge [6].

Evidence from orthopaedic series reports an expected postoperative haemoglobin fall of around 2-3 g/dL after primary hip or knee arthroplasty, with contemporary transfusion rates markedly lower than historical cohorts owing to tranexamic acid use and blood management protocols [7]. These data suggest that many postoperative haemoglobin checks do not result in intervention.

Several studies now challenge the need for routine postoperative blood tests in all patients undergoing primary total joint arthroplasty. A large retrospective study from China reviewed 395 consecutive elective THA cases and found that while 88% of patients exhibited abnormal postoperative results (including anaemia and hypoalbuminaemia), only 6.8% necessitated clinical intervention [8]. Moreover, a UK study of 353 elective THA patients reported that among those aged 70 or younger in the American Society of Anesthesiologists (ASA) grades 1-2, none required transfusion or experienced electrolyte disturbances; only 1.2% showed renal changes requiring minor intervention [9]. Specific risk factors, such as female gender, low body mass index, long surgical duration and low preoperative haemoglobin or albumin, were associated with an increased likelihood of intervention [8-11]. Hence, routine postoperative blood tests after total joint replacement (TJR) may not be necessary for every patient. This study arises from a need to align practice with modern care pathways and reduce unnecessary costs and delays within the UK setting. This study aims to determine whether routine postoperative biochemical tests after uncomplicated primary total hip or knee arthroplasty influence clinical management, discharge timing, or readmission rates.

## Materials and methods

This was a retrospective observational audit conducted at a District General Hospital in the United Kingdom at the beginning of 2023. Ethical approval was granted by the Trust's Audit Management and Tracking (AMAT) team, and the conduct complied with the ethical principles of informed consent, privacy, and anonymity. The retrospective, single-centre design may limit the generalisability of the findings to other settings. Broader, multicentre or prospective studies would be valuable to validate these observations and support wider application.

The study included a consecutive series of 99 patients who underwent primary total hip and knee replacement surgeries from January to March 2023. Inclusion criteria comprised consecutive adult patients who underwent primary THR or TKR and had postoperative laboratory tests performed. Patients were excluded if they had any bleeding disorder or if they became critically unwell intraoperatively and were transferred to a high-dependency or intensive care setting. The criteria ensured a consecutive cohort while minimising selection bias. However, ASA class, preoperative comorbidities, and intraoperative factors were not included in the criteria due to incomplete documentation, which is a limitation.

Demographic and clinical variables were collected from electronic medical records of the Trust, including age, sex, preoperative and postoperative haemoglobin, white blood count (WBC), platelets, serum electrolytes and creatinine levels, estimated glomerular filtration rate (eGFR), any subsequent lab, post-operative prescription, and reasons for delay in discharge and readmission. The timing of postoperative tests relative to surgery was standardised across patients, occurring within the first 24 hours after the operation. 

Statistical analysis was performed using IBM SPSS Statistics (version 26; IBM Corp, Armonk, NY) and Microsoft Excel (version 2016; Microsoft, Redmond, WA). Continuous variables were summarised as mean±standard deviation, and categorical variables were reported as frequencies and percentages. Cases with missing postoperative laboratory results were excluded from analysis. Normality of continuous variables and of paired differences was assessed with the Shapiro-Wilk test and graphically with Q-Q plots. A paired t-test was used for normally distributed paired data and the Wilcoxon signed-rank test when normality was not met. Statistical significance was set at two-tailed p<0.05 and effect estimates were presented with 95% confidence intervals.

## Results

**Figure 1:**
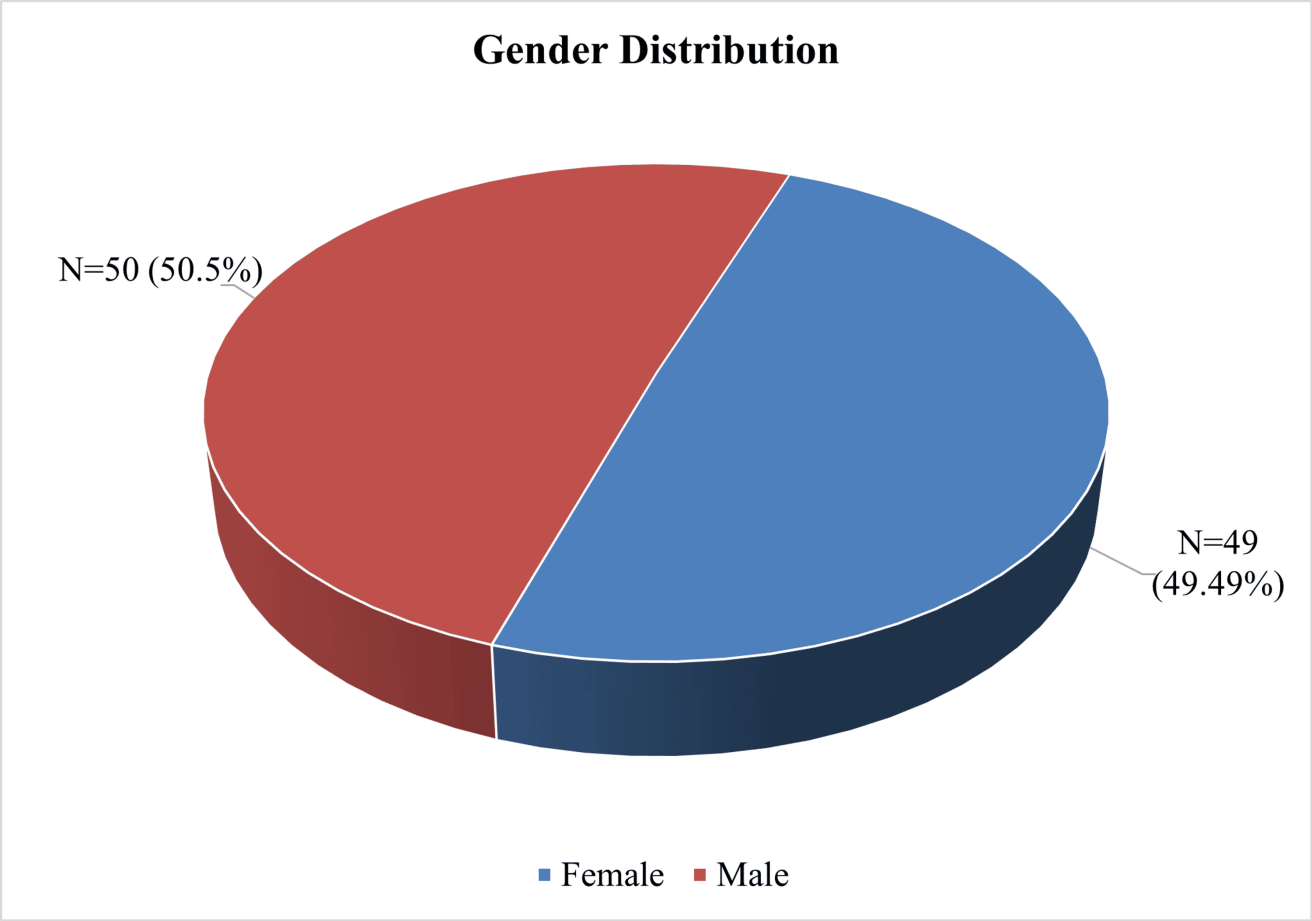
Gender proportion of participants in the observational study.

**Figure 2:**
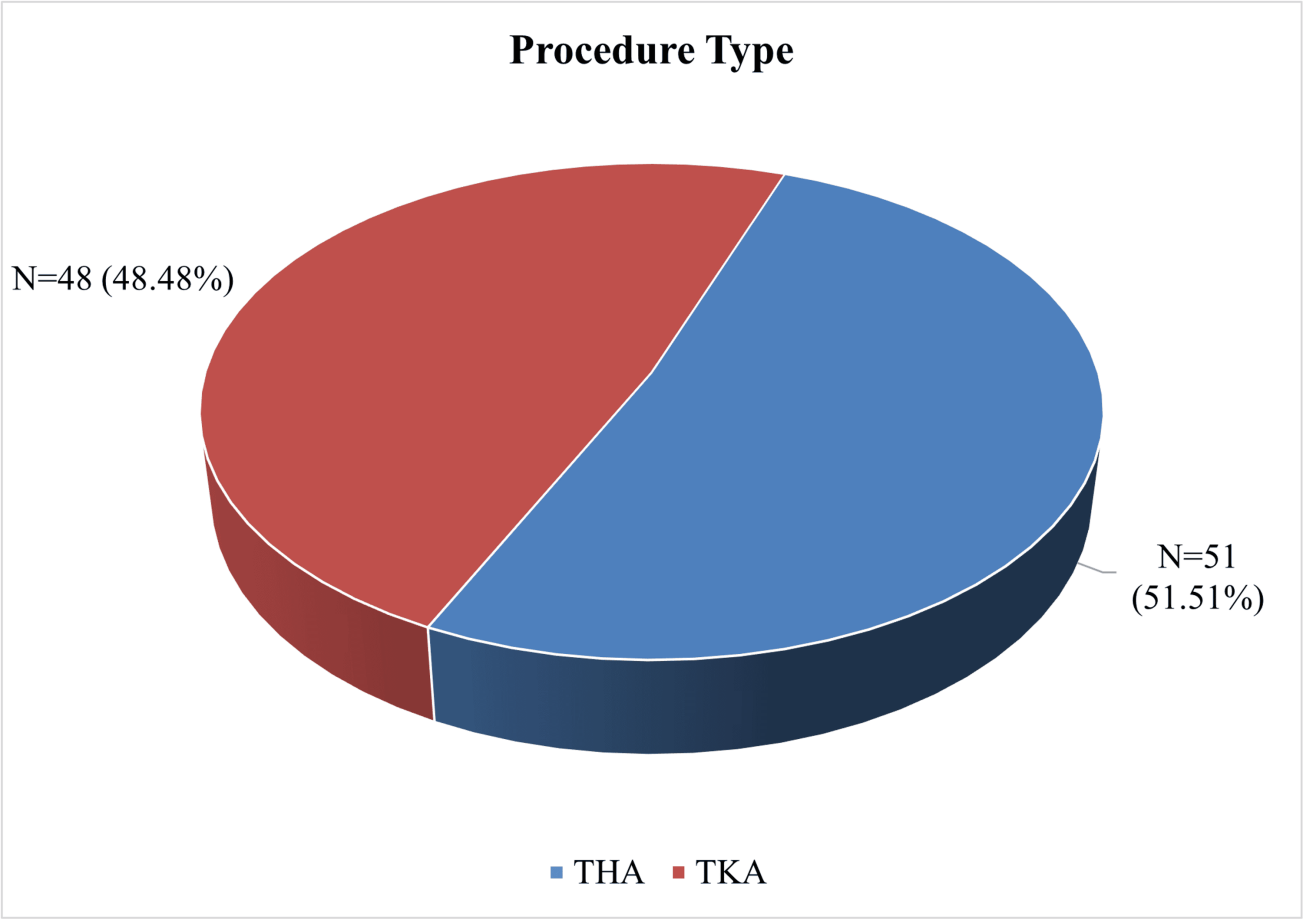
Procedure-type proportion of participants in the study TKA: total knee arthroplasty; THA: total hip arthroplasty.

A total of 99 patients were included in this study that underwent lower limb total joint arthroplasty. The mean age of the participants was 69.5±10.11 years. Of these participants, 50 (50.5%) were men and 49 (49.5%) were women (Figure 1). Based on the type of arthroplasty, 48 (48.5%) patients underwent total knee arthroplasty, and 51 (51.5%) patients underwent total hip arthroplasty, as shown in Figure 2.

Table 1 summarises the cohort’s laboratory changes (n=99). Haemoglobin fell from a mean of 136.33±12.83 g/L preoperatively to 115.81±13.03 g/L postoperatively, producing a mean decline of 20.52 g/L (≈2.05 g/dL). A paired t-test confirmed this fall was statistically significant (t=18.59, p<0.001). This magnitude of decline (≈2.5 g/dL) is consistent with expected blood loss after primary lower-limb arthroplasty. However, the mean postoperative value remains above common transfusion thresholds used in routine practice. White blood cell count rose from 7.03±2.20 ×10^9/L to 11.44±3.48 ×10^9/L, with a mean change of −4.41 ×10^9/L. This pattern is compatible with a normal postoperative inflammatory response. A paired t-test showed this increase was also statistically significant (t=−12.13, p<0.001), a pattern compatible with a normal postoperative inflammatory response. Platelet count fell from 276.41±73.63 ×10^9/L to 242.17±65.71 ×10^9/L (mean change = 34.24 ×10^9/L), with the paired t-test again reaching statistical significance (t=7.04, p< 0.001). The average postoperative platelet value remained within usual reference limits.

**Table 1:** Preoperative and postoperative laboratory parameters in THA and TKA patients (n=99) TKA: total knee arthroplasty; THA: total hip arthroplasty.

Variable	Pre-op (mean ± SD)	Post-op (mean ± SD)	Mean change (Pre − Post)	T-Value	P-Value
Haemoglobin (g/L)	136.33±12.83	115.81±13.03	20.52	18.59	0.000
White blood cells (×10^9/L)	7.03±2.20	11.44±3.48	−4.41	−12.13	0.000
Platelets (×10^9/L)	276.41±73.63	242.17±65.71	34.24	7.04	0.000

Although these changes were statistically significant, they rarely altered clinical management. Only nine patients (9.1%) had postoperative haemoglobin below 100 g/L and no patient required a transfusion. One patient was symptomatic with light-headedness, following a drop in haemoglobin from 130 to 97 g/L. The patient was observed and discharged on day 3. Another patient sustained a large fall in haemoglobin from 134 g/L preoperatively to 79 g/L postoperatively, accompanied by hypotension and wound bleeding. Treatment with tranexamic acid, intravenous fluids and strict input/output monitoring stabilised the patient. Repeat bloods later showed haemoglobin 99 g/L and blood pressure had normalised, and discharge occurred on day 5.

With regard to renal function and electrolytes both before and after surgery, 93 patients (93.93%) had normal eGFR and creatinine levels both pre-op and post-op. The remaining five of them (5.05%) had abnormal eGFR both pre-op and post-op, but no action was undertaken. One patient had eGFR dropped to 39 (stage 3 chronic kidney disease (CKD)) from 84, treated with IV fluids and discharged on day 12. The other patient was known to have stage 5 CKD, so renal input was sought while he was an inpatient, nephrotoxic drugs were stopped, enoxaparin was reduced, and IV fluids were given. He was discharged after two weeks.

A total of 91 (91.91%) patients had normal serum electrolyte levels. The non-normal entries included eight patients (8.08%) who had abnormal Na^+^/K^+^, out of which four of them had fluid restriction, and the other four did not receive any treatment. Repeat Na^+^ levels were marginally better in all eight patients, and there was a delay in the discharge of all these patients.

**Figure 3:**
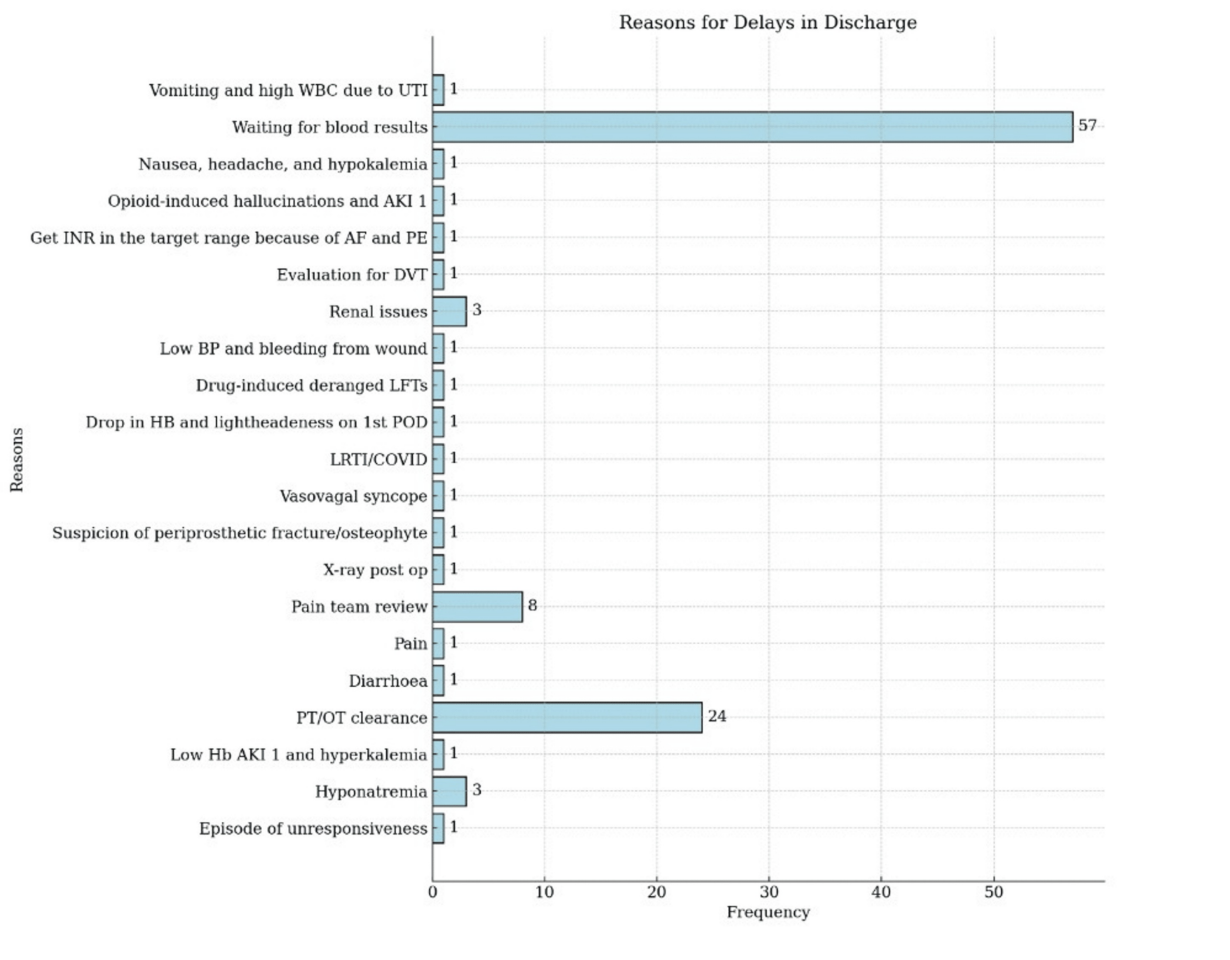
Reasons for delay in discharge

The reasons for delaying the discharge are mentioned for the patients, of which 57 (51.35%) were due to waiting for blood test results, 24 (21.62%) due to clearance of patients from operation theatres and several other reasons presented in Figure 3. The results suggest that the most informative preoperative risk factors are lower preoperative haemoglobin, lower baseline renal function (eGFR/raised creatinine) and electrolyte abnormalities or ongoing diuretic use. Those factors commonly predict which patients required fluid therapy, renal input, electrolyte correction or closer observation.

Regarding readmission, a total of nine (9.09%) patients were readmitted. The recorded readmissions related mainly to local wound problems, postoperative limb swelling or pain suspicious for venous thromboembolism, surgical site infection and one fall with minor injury. Most cases involved clinical assessment, imaging (D-dimer, Doppler ultrasound) and targeted management such as antibiotics, wound care or later operative treatment for stiffness. The events that prompted readmission appear to be clinical problems that would not have been prevented by blanket postoperative testing. That finding supports a selective approach in which routine laboratory testing is reserved for patients with preoperative risk factors or new clinical concerns.

None of the patients required blood transfusions, despite a minority developing notable laboratory abnormalities. Most abnormalities were managed conservatively through observation, fluid modification, or brief electrolyte therapy, and improved before discharge. Hence, it can be said that routine postoperative testing seldom altered immediate management in this cohort, with the majority of patients showing normal laboratory results at discharge. Five patients were excluded due to incomplete biochemical data.

## Discussion

The study aimed to evaluate the clinical utility of routine postoperative blood tests after primary lower-limb arthroplasty. The findings revealed that routine postoperative testing produced predictable, largely non-actionable changes in laboratory indices (mean haemoglobin fall ≈20.5 g/L). No one required a transfusion, and most postoperative results were either normal or abnormal but clinically self-limiting and managed conservatively. Preoperative anaemia, deranged electrolytes, and impaired baseline renal function were the factors most commonly associated with inpatient management or further investigation.

Our haemoglobin results matched contemporary reports of an average fall of about 2-3 g/dL after primary hip and knee replacement, but with very low transfusion need [7]. Contemporary transfusion rates after primary THA/TKA have fallen dramatically with routine tranexamic acid (TXA) and restrictive thresholds [2]. Several multicentre and single-centre series have reported similar mean haemoglobin drops alongside negligible transfusion rates in the era of tranexamic acid and modern blood-management pathways [7-10]. Our finding of zero transfusions, despite a statistically significant haemoglobin drop, is in line with the literature. A study detected a very low transfusion rate (0.8%) after TKA/THA, and concluded that haemoglobin/haematocrit measurements drawn immediately post-surgery rarely influenced management for low-risk patients [12]. However, a study reported a high rate of abnormal postoperative laboratory findings and a notable need for clinical interventions among patients who underwent THA for hip fracture in a semi-urgent setting [13]. This suggests that routine postoperative testing remains important for individuals with specific risk factors. Halawi et al. [11] reported that routine postoperative labs rarely altered care and argued for selective testing based on preoperative risk, for example, low haemoglobin, advanced age, and medical comorbidity.

Several large cohorts reinforce the low clinical yield of blanket testing. Recent evidence regarding routine labs after primary arthroplasty synthesised multi-centre data showing that only a small fraction of patients need any intervention for postoperative abnormalities and concluded that abnormal values were predictable from pre-op status and selective strategies are safe and cost-effective [4, 14]. Electrolyte and renal results in our series were also aligned with recent evidence. Selective-testing frameworks show that postoperative sodium/potassium disturbances are uncommon, typically mild, and concentrated in patients with pre-existing derangements, diuretic use, or perioperative fluid shifts. When they occur, they infrequently require more than brief fluid/electrolyte management [11,14]. In our cohort, ≈92% had normal electrolytes and ≈94% had normal eGFR/creatinine post-op. The few abnormalities were managed conservatively, and none escalated to dialysis, ICU, or invasive intervention.

Crucially, our readmissions were not driven by low haemoglobin, leukopenia, thrombocytopenia, acute renal failure, or refractory dysnatraemia/dyskalaemia. Rather, they reflected common post-arthroplasty issues, leg swelling with deep vein thrombosis (DVT) ruled out, wound oozing without infection, cellulitis/surgical site infection (SSI) treated with short-course antibiotics, falls without fracture, or postoperative stiffness [15], conditions that cannot be prevented by routine next-day lab panels. This agrees with the broader literature showing that routine labs seldom predict or prevent the predominant causes of early readmission after uncomplicated THA/TKA (pain, wound concerns, swelling, mobility issues), whereas clinical assessment and pathway adherence matter more [16].

In terms of predictors, actionable abnormalities include lower pre-op haemoglobin/albumin, older age, higher ASA class, longer operative times and greater blood loss, or baseline electrolyte derangements [11]. According to Li et al., identified risk factors include age, ASA≥III, female sex, low pre-op haemoglobin and baseline electrolyte issues [14]. These are consistent with our observation that the most informative risk factors in practice were low pre-op haemoglobin, diminished renal reserve, older age, and deranged electrolytes/diuretics, precisely the features that prompted fluids, renal input or electrolyte correction in a minority of patients. Hence, for clinically stable, low-to-moderate-risk patients undergoing uncomplicated primary THA/TKA, selective postoperative laboratory testing, guided by preoperative risks, intraoperative course, and clinical signs, appears safe. Routine panels for every patient added delay and cost, while rarely changing management. In the National Health Service (NHS), routine postoperative blood tests cost approximately £12 per patient [4], which means £1200 can be saved per 100 patients.

The retrospective design meant reliance on existing medical records, which may have introduced selection bias, under-reporting or inconsistent data capture [17]. For example, the absence of ASA classification and detailed comorbidity data limits the ability to stratify risk and, therefore, they should be included in future analyses for improved risk-adjusted conclusions. The single-centre setting and modest sample size constrain external validity. Cost implications and patient-reported outcomes were not assessed, which reduces understanding of the wider organisational and clinical impact. Lack of data on patient satisfaction and anxiety related to prolonged stay may have obscured important harms or benefits associated with selective postoperative testing. Future studies should include cost-effectiveness analysis, patient outcomes, and patient satisfaction, alongside evaluation of clinical endpoints such as readmissions, transfusions, electrolyte correction and impact on length of stay. Moreover, a prospective, multicentre validation of a risk-stratified testing algorithm would strengthen the evidence for selective testing.

## Conclusions

Routine postoperative laboratory testing following uncomplicated primary THA and TKA rarely alters clinical management and often identifies abnormalities of limited significance. No patient required a blood transfusion, and readmissions were driven by common clinical issues such as wound problems, swelling, or stiffness rather than laboratory abnormalities. The findings support a selective, risk-based approach, with testing reserved for patients presenting with factors such as low preoperative haemoglobin, renal impairment, electrolyte derangements, and advanced age. Future work should aim to develop and validate a practical model incorporating preoperative haemoglobin, renal function, ASA status and age to guide testing decisions in routine practice. The adoption of ERAS protocols and the routine use of tranexamic acid further reduce transfusion requirements, strengthening the case for targeted testing. Implementation of a risk-stratified strategy could reduce unnecessary costs and delays in discharge without compromising patient safety. For practice in similar healthcare settings, postoperative labs should be guided by preoperative assessment and clinical indications rather than performed routinely for all patients. 
